# Texas State Medical Association

**Published:** 1876-05

**Authors:** 


SOUTHERN MEDICAL RECORD.

VOL. VI. MAY, 1876. NO. 5

### Original Communications.

### TEXAS STATE MEDICAL ASSOCIATION.

By A. R. K.

We have received a copy of the TRANSACTIONS of the Texas State Medical Association, in its Seventh Annual Session, at Austin city, in the State Capitol, on the 6th, 7th, 8th, and 9th of April, 1875.

It is a source of pleasure to us to witness from year to year the increased activity of our professional brotherhood, and mark their constant improvement, and the zeal with which they urge forward the car of progress far away on the outskirts of the nation. Organized action and co-operative movement are the best means to secure our purpose of placing our profession on a firm and lofty basis. County Societies and State Associations are producing wonderful results, and we hope the day will soon come when such bodies will be efficiently working in all our broad land.

The Association was welcomed to the capital in a very neat address by Dr. W. A. Morris, Chairman of the Committee of Arrangements and member of the Travis county Medical Society, on behalf of said body. Dr. A. G. Clopton, of Jefferson City, President, called the Association to order, and prayer was offered by Rev. Mr. Lee, of St. David's Church. Fifty members responded to roll call, about fifty more were admitted to membership during the session, so the Association must have about one hundred and fifty members, embracing men from nearly every part of Texas. Harmony and cheerfulness seem to have held full sway during the entire session.

During the session the members of the Association and other visitors to the city were hospitably entertained by the citizens; and dinners, suppers, balls, and social parties were given them by Gov. Coke, the Travis County Medical Society, Dr. M. A. Taylor, U. S. A., and Mr. G. B. Zimpleman. They visited the Asylums, and other public institutions about Austin, under the care of Dr. D. R. Wallace.

The report of the transactions is embraced in a pamphlet volume of 223 pages, neatly printed at the office of the *Colorado Citizen*, Columbus, Texas; Dr. R. H. Harrison, chairman of the Committee of Publication. This Committee complain of the almost illegible penmanship of some of the articles contributed by the members of the Association, and there are several errors of grammar and composition which should not be seen in communications coming from members of a learned body like this. Some of the articles were so difficult to decipher, and blurred with so many errors, that the Committee laid them aside. This fault, we regret to say, is too generally prevalent in the profession, and while we are willing to admit that the basilar branches of orthography, etymology and penmanship do not make a physician any better practitioner, yet their lack may greatly curtail his usefulness otherwise, especially in imparting his knowledge, and derogate from his status as a man of learning. In this volume we detect some instances of faulty grammar and slovenly composition, as well as incorrect spelling, especially of medical terms, and even names of authors.

The Association adopted the system of ethics issued by the United States Medical Association. The sum of five dollars is required to be paid upon admission to membership, and annually thereafter.

A remodeled form of constitution was adopted, with by-laws after the style of those used by other State Associations and parliamentary bodies.

The Secretary is paid $200 *per annum* for his services, and Dr. W. A. East was chosen to serve for five years.

Five Standing Committees, composed of three or five members each, are appointed annually, viz:

1. Committee on Science and Progress of Medicine.
2. Committee on Publication, Finance and Claims.
3. Committee on Record of Cases.
4. Committee on Indigenous Medical Resources of the State.
5. Committee on Arrangements and Invitations.

The Secretary reports the collection and disbursement of nearly four hundred and fifty dollars for the year 1874.

Many resolutions were offered and adopted each day of the session, among which we select one recommending and urging the organization of county Medical Societies in every county of the State, and for them to present to this Association every year a report of the membership and proceedings; one urging upon the people generally and the State authorities the value and necessity of observing and enforceing sanitary and hygienic measures and protection against infection and epidemic diseases; one to prepare and present to the Centennial meeting in Philidelphia, a history of the science and progress of medicine, especially that part that pertains to the interests of Texas; one to report at next meeting on the anatomical and physiological difference between the white and negro races; on the modifications of their respective diseases, and the difference in treatment resulting therefrom; one recommending joint action of the seaboard States in securing the establishment of a National Board of Health.

Dr. T. M. Blackemore, of San Marcos, Hays county, reported the case of a fœtus whose occipital bone was absent, the scalp *in loco* being perfectly cicatrized, as though the occiput had been (spontaneously) amputated; the funis was only nine inches long, about an inch and a half in diameter, and contained nearly all of the intestines; also, he presented a three months fœtus with membranes and liquor amnii entire, after being cut from the placenta.

The chairman of the Committee on Indigenous Medical Resources, Dr. D. F. Stuart, of Houston, after reciting the difficulties incident to the collection of material for a report, submitted a catalogue of one hundred and sixty botanical agents as indigenous to Texas. Among these we notice *Tephrosia Virginiana*, or Devil's shoestring, as a remedy for stings of insects, bites of poisonous serpents, centipedes, etc. The virtues of this plant were brought to our notice two years ago, by our assistant editor, Dr. A. R. Kilpatrick, of Navasota, Texas; the *Scutellaria Lateriflora*, which has the reputation of curing hydrophobia. Of the mineral resources, Dr. S. says Texas can boast a supply not inferior to any land; and, beginning with gold, silver, copper, iron and lead, he gives a list of the minerals, salts and alkalies; together with chalybeate, sulphur, salt, and acid springs, lakes and wells enough to cure all the ills to which flesh is heir. These resources, herbs, minerals, and health-restoring springs are not confined to certain localities, but scattered bounteously over all the State.

Dr. Dubose brought up the question of the virtues of the so-called "Mad Stone." After some discussion, the conclusion was reached that it is no better than a cupping-glass.

Dr. H. Ryan presented the form of a "bill" to be laid before the next Legislature, regulating apothecaries and druggists in the sale of poisonous drugs, and prescribing their punishment in case of infraction of said law. Also, one regulating the taxes on venders of nostrums and patent medicines.

Dr. J. A. Gibson, of Richmond, Fort Bend County, offered preamble and resolutions on the very important subject of liens upon crops for land rent and supplies and advances furnished to aid in their production; setting forth the injustice done physicians by this class legislation, whereby we are robbed of our fees and cut out of compensation for our daily labor. The President of the Association and the Executive Committee are charged with the duty of bringing this matter before the next Legislature, and urging the amendment of the laws so as to secure to physicians the payment of a reasonable compensation for medicines and professional services. It is matter of surprise to us that our legislators and statesmen are crying out so lustily against *class legislation*, when these lien laws are nothing else. Every medical practitioner in our broad land suffers the loss of hundreds of dollars year after year in consequence of the landlord, or the merchant, swindling the operative out of his year's labor and crop, under the cloak of the lien law. It is verily a *lean law* for the doctor.

Dr. T. J. Heard, of Galveston, offered a resolution to ask the State government to appropriate five thousand dollars for the culture of the *eucalyptus globulus*, the money to be controlled by the Medical Association. He also offered a prize of one hundred dollars for the best essay on the *eucalyptus globulus*.

Dr. J. A. Gibson, of Richmond, propounded the query: "Would, or would not, emasculation be advisable in the treatment of insanity induced by self-abuse? The question was discussed at some length without any result.

Dr. H. W. Brown, of Waco, said the best way to suppress charlatanry, imposture and the use of patent medicines, was to have Medical Societies in every county and large city throughout the land, and that every regular physician should be *compelled* to unite with these organizations, and when any one was proven unfit and unworthy of membership, he should be expelled ignominiously. We doubt if this would operate as a cure for this grievance.

Twenty-one physicians from all parts of the State were appointed by the president as delegates to the American Medical Association, at its next session in Philadelphia.

Having disposed of the operative procedures of the Association, we now come to a review of the president's address. Merely glancing at the struggles of the medical art in early times, he made a sweeping and rapid sketch of the different causes which have operated in producing the extraordinary vicissitudes, so eminently characteristic of its early history; showed that its progress towards its present advanced state has been gradual, sometimes arrested by the caprices, prejudices, superstitions and knavery of mankind, so lamentably prevalent during the dark and barbarous ages. He alludes with pleasurable emotions, to the later and brighter features in the history of our noble science, and its glorious disenthrallment from the withering influences which so long held it under bondage. All the great improvements and discoveries, especially those reflecting such efflulgent glory on the present generation, are called up in rapid review, and their marvelous influences upon our noble profession expressed in thoughts that breathe and words that burn.

"The warrior, the statesman, the lawyer — each have their reward in plaudits, preferment and worldly honor; while the physician, though unrewarded with fame, (only among his fellow-laborers in the science), stands in his relation to society higher than either. He may pass from the stage of action poor, obscure and unknown ; yet, if he has performed his duty, if he has devoted his intellect to the task assigned him, he has benefitted his race, and will receive his reward — he has not lived in vain.

"The spirit which inspires the physician should be neither sordid or selfish. He must have a heart to love and sympathize with humanity, and an intellect cultivated and disciplined to duty. Then let us divest ourselves of all envy ; let no selfish disappointment come between us and this association, and let us labor together in harmony and social good-will for the common advancement and elevation of our noble profession."

The next article which claims our attention is the "Report on the Science and Progress of Medicine," by Dr. D. R. Wallace, of Austin, chairman of committee on same. The paper covers twenty pages, and gives quite a lucid review of theories and practice of medicine for the last hundred years. He contends that what differences, discrepancies and changes have taken place in this space of time are traceable partly to fashion, partly to the influence of authorities, and partly to the necessities of the case and the changes in the manner of living of the people, and the character of diseases. Hence the *desuetude* of bleeding, emetics and drastic purgation. We durst not charge our illustrious predecessors with ignorance, charlatanry, or routine practice, seeing that they were successful in combating disease, and were judged worthy of praise and honor by the illustrious and intelligent of their day. He deals out scathing rebukes for the hot haste of our people in establishing so many medical schools and yearly throwing out thousands of graduates with no preparation at all adequate to the discharge of their arduous duties and profound obligations. They are mere fungi, parasites upon society; *fruges consumere nati*, and contributors in no sense to the public weal. While our little cross-roads medical colleges annually grant diplomas to three thousand men, the German Empire, with a population of forty-two millions, has only nine medical schools, and in 1874 graduated only three hundred and sixty-nine; a proportion of a little over one to ten of ours.

The next paper is by Dr. John H. Pope, of Marshall, chairman, on "*Climatology and Epidemics*." He begins by lamenting paucity of data, material and statistics, which he expected to be contributed by professional co-laborers in different parts of the State. For a full and satisfactory report to be made on the subject, there should be suitable persons appointed in every county, equipped with an outfit of meteorological instruments and other apparatus to take observations and keep records of them. Let the county medical socities have sanitary committees to observe and report monthly the condition of the health of the county. The report goes on to show that during the year 1874, Texas was quite healthy, there being no epidemic anywhere, although the high temparture and the amount of rain were calculated to produce disease. The thermometer at Marshall indicated ninety-three and a half degrees as the maximum in June, ninety-six degrees in July, ninety-nine degrees in August, and ninety-two in September.

Dr. R. H. Harrison, of Columbus, chairman, furnishes a report on *The Epidemics of* 1873 *in Denison, Calvert and Columbus*. This disease, which killed so many in the towns named, as well as elsewhere, was considered genuine yellow fever by many physicians, as well as by laymen who had seen yellow fever in other years and places. This report, however, does not admit it was yellow fever, but a continued fever, or a hœmorrhagic malarial fever. Dr. H. addressed a printed circular to such men as he thought were qualified to report on the fever which prevailed at those places, but their answers were so discordant or contradictory that they suggest reflections too humiliating to our profession to be indulged. The history of the epidemic shows that it spread from Shreveport, La., to Calvert and the other towns, the formites being carried by individuals. It is to be feared that personal interest and the interest of corporate bodies has had much to do in preventing a full and candid examination and history of the disease being made. It is well known that the great dread of epidemics, especially of yellow fever, deters thousands of people from visiting and settling in places where such disease is liable to appear and spread. The report winds up by casting doubt on the epidemic yellow fever of 1873, and even says the question is far from being settled whether the yellow fever was anywhere in Texas in 1867, but call it "*Texas Yellow Fever*;" saying, moreover, that "the question is of importance as affecting the fair fame of our State."

Dr. T. J. Heard, of Galveston, chairman, reports on *Malarial Hœmaturia*, in a very interesting paper, covering twenty-four pages. Deeming a comparison of views on this subject desirable, he sent to several physicians a printed circular, embracing interrogatories covering the main points: such as, the kind of seasons in which it prevails; the influence of drainage; prior condition of patients; social condition and manner of living of patients before and since the war; influence of alcoholic drinks; what influence has the customary mode of medication; whether there is an inflamed or hyperemic condition of the duodenum; to what extent were the kidneys involved? Out of a great number addressed, he received seventeen replies, some of them quite brief, almost monosyllabic. We summarise the answers, and find that Malarial Hœmaturia prevails only in highly malarious localities; that lack of drainage has little agency in causing it; rain and moisture, together with high range of temperature, have much to do in producing it; it prevailed to a limited degree before the war and much greater since, and poor, coarse, heavy, indigestible food is a factor of considerable influence; it occurs as often in those who do not drink liquor as in drunkards, but these latter are more liable to die. It generally attacks those who have been debilitated by disease, especially intermittents, and such patients are most apt to die. It is not thought that the use, or abuse, of quinine causes the disease. The duodenum is tender and painful in most cases, while the main force of the malady acts on the kidneys and liver, producing toxœmia and enuresis. Dr. H. contends that t is disease is as much the result of the disuse of emetics and cathartics, and the practice of giving large and repeated doses of quinine without first cleansing out the stomach and rousing the portal system, as it is of malaria or unsuitable food. In treating malarial fevers, which recur every week or two weeks, he advises the use of preventive medicines, to be given on the *day before* the expected return. A favorite recipe with him is :

| | | |
|---|---|---|
| R | S. Quinine | gr. i. |
| | S. Ferri | gr. iij. |
| | S. Magnes | oz. iij. |
| | S. Acid, dilute | oz. jss. |
| | Tinct. Zinziber | oz. ss. |
| | Aqua Fort | q.s. to make 8 oz. |

Dose, one teaspoonful in half a tumbler of water three times a day.

A monograph by Dr. E. P. M. Johnson, of Marshall, on the same disease, in which he traverses nearly the same ground with Dr. Heard. There is some variation in the course of treatment ; he uses diuretics, antacids and acidulous drinks alternately ; mustard externally, and quinine in decided doses until the full influence is obtained. He extols the use of lemonade and sul. magnesia with ice. Keep the bowels open, the skin and kidneys acting ; nourish your patient and keep him under the influence of quinine, and he will recover.

Dr. O. H. Seeds, of Columbia, reports two cases of *Traumatic Tetanus*, one in a girl aged fifteen ;—the tetanus was well marked, and lasted thirty-one days, during which time she took 2,720 grains of chloral and one ounce of bromide of potash, which controlled the spasms and cured the case. The other was in a woman, aged twenty-eight, and pregnant at the time. The same medicines were used until bromisar developed, when the bromide was dropped, the chloral being continued alone. The case recovered slowly, slight symptoms continuing five weeks. The chloral had no deleterious effect on her generative organs, or the fœtus.

Dr. W. J. Burt, of Austin, reports a case of *Hydrophobia*, occurring in a little girl, who was bitten while playing with a pet shepherd-dog, which was not then suspected of being rabid, but subsequently it was satisfactorily found out from his biting other children, and his strange, wild conduct The girl was bitten on the 1st of October, 1874 ; the Doctor was called to her the 10th of December following, and she died on the next day, after suffering with the usual symptoms of the disease. Chloral was used freely; it subdued the convulsions, and she died calmly.

Dr. B. C. Hadra, of Austin, gives five cases of *Trichiniosis*, which came under his care in 1874, two of which died. He does not pretend to any originality in the treatment, but refers to the authors of the day. The cases are interesting, however, from the fact that trichiniosis rarely occurs in the South.

*Pschycological Medicine* next claims attention under the pens of Dr. J. H. Sears, of Waco, and Dr. J. J. Burroughs, of Houston, both of whom treat the subject rather lightly and cavalierly, and probably we had as well pursue the same course. Both seem to have studied the subject well, and give very fair expositions of the prevalent opinions entertained upon the subject.

Dr. T. D. Wooten, of Paris, Lamar county, chairman of the committee, has given a lengthy *Report on Surgery*, and we regret that our space will not allow us to give such an extended notice as the article deserves. Inflammation, septicœmia, wounds, disease of joints, club-foot, stricture, aneurism, surgery of the rectum, torsion, Esmarch's bloodless operation, anæsthetics, aspiration, laryngoscopy, ovariotomy and gastrotomy are all treated of at some length, and show much research and reading, as well as practical knowledge.

Dr. M. A. Taylor, U.S.A., Austin, gives an essay on *Esmarch's Method of Bloodless Operation*, and details five cases in his practice, where he had employed the method ; and he says the bandage produces the local anæsthetic condition almost equal to chloroform locally applied. He considers this method one of the most valuable of the surgical expedients of modern times.

Dr. J. Cummings reports some interesting *Cases in Surgery*. Dr. W. H. Park, of Tyler, also reports *Cases in Surgery*: operations for staphyloma and cataract.

Dr. G. W. Trueheart, of Galveston, reports a case of *Skin-grafting* and transplantation of skin for relief of scars following burns, etc. In one case, he transplanted a piece of intigument from the chest in the palm, and the boy has now the phenomenon of hairs growing in the palm of his hand.

Dr. Hillary Ryan, of Austin, reports a case of *Carcinoma and Stricture of the Rectum*; the patient died and a *post-mortem* was not permitted; also, a case of *Lithotomy*.

Dr. Greensville Dowell, of Galveston, reports *Cases of Stricture of Urethra*, and introduced instruments of his own devising, which he considers superior to others. He also reports on a new method of operating for the permanent cure of *Hernia*, by a subcutaneous stitch made with a large crescentic needle having the eye in the middle, similar to the horse-collar needle. We have heard of his operation, and he reports eighty cases operated on, sixty-eight of which were cured. The Doctor is ingenious and industrious, and is destined to exert a beneficial influence throughout Texas.

Dr. O. H. Seeds, of Columbia, reports on *The Therapeutic Use and Abuse of Gossypium Herbaceum*, but says nothing in its favor. It is considered uncertain, unsafe and not worthy of being adopted into the list of regular medicines. It produces copious hœmorrhages sometimes, and when given in large quantities will cause abortion. Its emmenagogue powers are *nil*.

We have used it in a few instances as an oxytocic, in decoction, when it was followed by violent uterine pains and contractions, and think it has a good deal of power that way.

